# Atraumatic Spleen Rupture: A Rare Fatal Complication of Dengue Hemorrhagic Fever

**DOI:** 10.7759/cureus.95114

**Published:** 2025-10-21

**Authors:** Harsha Vardhini, Magesh Murali

**Affiliations:** 1 Critical Care Medicine, St. John's Medical College Hospital, Bengaluru, IND

**Keywords:** atraumatic splenic rupture, conservative vs surgical management, critical hemorrhagic shock, dengue complication, dengue hemorrhagic fever (dhf), massive blood transfusion, splenic arterial embolisation, tropical fever

## Abstract

Dengue is a common tropical infection in Southeast Asian countries, with clinical severity ranging from a self-limited acute febrile illness lasting 3-5 days to severe disease with a wide spectrum of complications. These complications can include extensive shock, coagulopathy, hemorrhage, multi-organ dysfunction, and death.

In this case report, we describe two cases of atraumatic splenic rupture occurring in the context of dengue hemorrhagic fever. In these two patients, non-traumatic rupture of the spleen occurred in the absence of any external injury, with positive dengue signs and serology. The major clinical challenges in managing these patients were hypovolemic shock, thrombocytopenia, and coagulopathy. Both cases were managed non-operatively, with an emphasis on organ preservation.

The first patient responded well to aggressive resuscitative efforts, was closely monitored for clinical deterioration, and was eventually discharged home in stable condition. The second patient presented with cardiac arrest and required intensive management, including invasive ventilation, massive transfusions, continuous renal replacement therapy, and splenic artery embolization to control bleeding. Despite these interventions, he unfortunately succumbed to the illness.

Effective source control is a critical component of resuscitation in hemorrhagic shock, but the decision to intervene surgically or conservatively must be individualized. Although both patients had similar baseline characteristics, except for differences in their hospital presentations, the choice between a more conservative approach and a liberal approach, such as interventional management, depended on their respective clinical responses during resuscitation.

## Introduction

Dengue fever is a common arthropod-borne viral infection caused by the Flavivirus genus. It is a single-stranded RNA virus with four distinct serotypes: DENV-1, DENV-2, DENV-3, and DENV-4 [1]. Dengue remains a significant public health concern, with nearly 5.9 million cases reported globally in 2019, predominantly affecting populations in Southeast Asian countries [2]. The disease is transmitted to humans through the bite of the female Aedes mosquito, which breeds in man-made water reservoirs typically found at the household level. After an extrinsic incubation period of 8 to 10 days within the mosquito, the virus becomes transmissible, and the intrinsic incubation period in humans ranges from 4 to 7 days.

The pathogenesis of dengue involves direct viral injury and immune-mediated cellular damage, leading to endothelial dysfunction, macrophage activation, and platelet destruction. These processes result in vasculopathy, coagulopathy, cytopathy, and organ dysfunction, collectively defining severe dengue [3].

Dengue hemorrhagic fever with shock is one of the severe manifestations of the disease. Common bleeding manifestations in dengue include epistaxis, hematemesis, melena, hematuria, and menorrhagia, with intracranial and retroperitoneal hemorrhages being rare [4].

Splenic involvement with subcapsular hematoma is an uncommon complication, reported in approximately 1.5% of all dengue cases [5,6]. Due to the rarity of the complication, it is difficult to diagnose unless clinicians have a very high index of suspicion. Only a few case reports describe this rare presentation and hence, there is no clear consensus or guidelines to approach this complication.

In this report, we present two cases of spontaneous splenic rupture secondary to dengue-associated coagulopathy. Both patients presented to the emergency department with clinical features consistent with severe dengue. While dengue fever complicated by coagulopathy and hemorrhagic shock is not uncommon, splenic rupture in the setting of severe dengue is a rare clinical presentation [5-7]. We highlight the challenges encountered and the management strategies implemented following complex clinical decision-making. Management differed based on clinical progression: one case was managed conservatively with close monitoring, while the other required definitive intervention with splenic artery embolization.

## Case presentation

Case 1

A 33-year-old man with no known comorbidities presented to the Emergency Department with a five-day history of fever associated with chills, headache, myalgia, and abdominal pain localized to the left upper quadrant for one day. He was previously evaluated at an outside facility and found to be dengue NS1 positive. The patient was triaged to the red zone of the Emergency department as his National Early Warning Score 2 (NEWS2) score was 13 [8]. On examination in the Emergency Department, the patient was tachycardic (110 beats per minute), hypotensive (89/60 mmHg), and had an elevated lactate level of 4.1 mmol/L. Abdominal examination revealed severe tenderness in the left hypochondrium with diffuse guarding and rigidity. Point-of-care ultrasound showed free fluid in the splenorenal space and a perisplenic collection.

**Figure 1:**
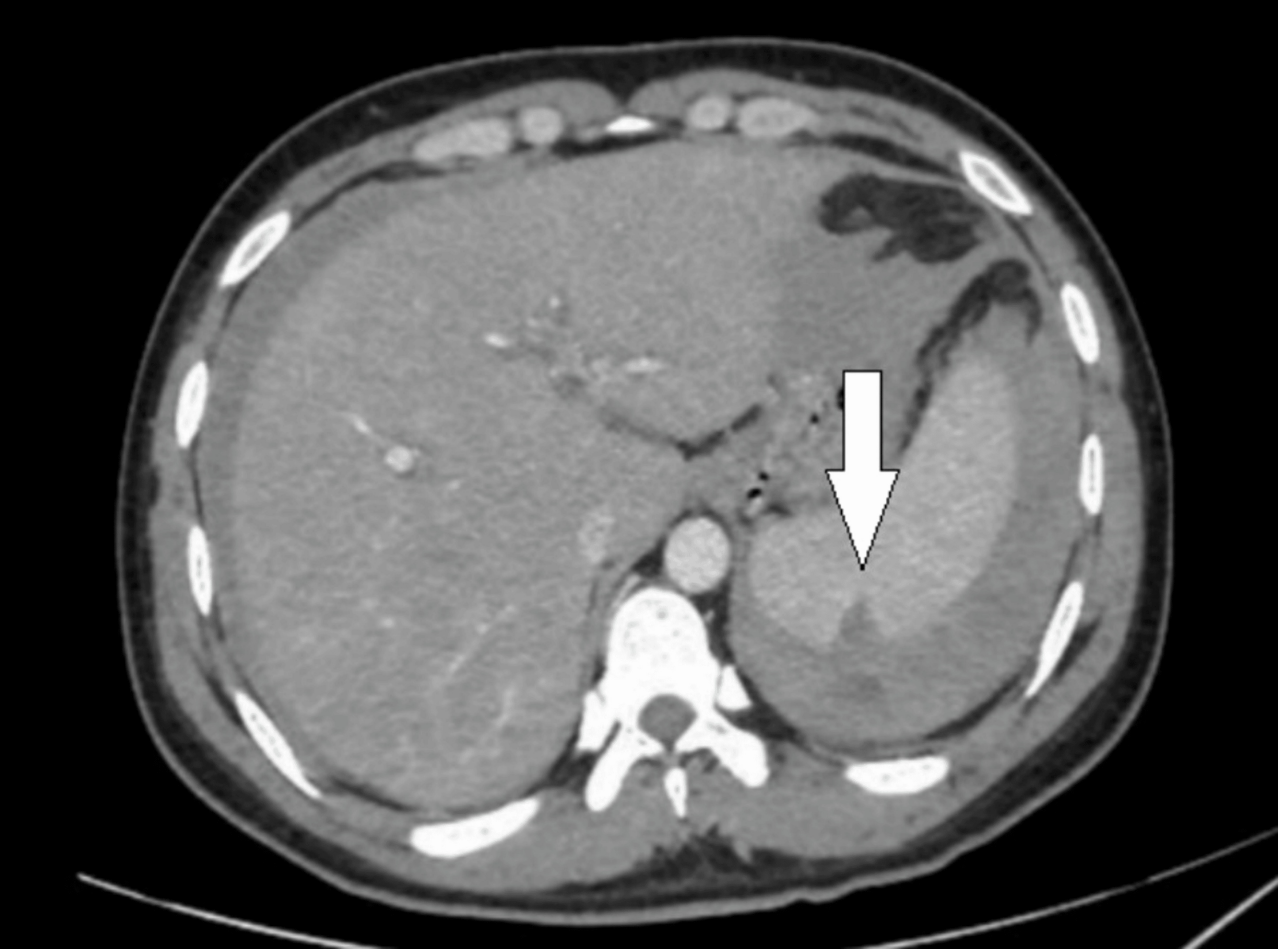
CECT abdomen on day 1 CECT: Contrast-enhanced computed tomography

A contrast-enhanced computed tomography (CECT) scan of the abdomen revealed splenomegaly with a 1.7 cm splenic laceration, a subcapsular hematoma measuring 8.3 × 2.6 × 2.9 cm, and hemoperitoneum. Additionally, hepatomegaly and pleural effusion were noted (Figure 1). The patient was stabilized in the Emergency Department. Due to the need for intensive monitoring and anticipation of deterioration, he was admitted to the intensive care unit (ICU) for further management.

In the ICU, he was supported with non-invasive positive pressure ventilation, bilevel positive airway pressure (BiPAP), due to respiratory distress. Early pulmonary edema secondary to fluid resuscitation and blood transfusion versus acute respiratory distress syndrome (ARDS) were considered as differentials for the respiratory distress. The patient presented with B-lines and a minimal pleural effusion on a point-of-care ultrasound (POCUS) examination in the ICU. Fluid administration, blood transfusion, and vasopressor support were optimized carefully after regular hemodynamic assessments of the patient. Initial laboratory investigations revealed thrombocytopenia, elevated liver enzymes, and increased ferritin levels (Table 1). Following the discussion of the multidisciplinary team consisting of an intensivist, a surgeon, and an interventional radiologist, conservative management of the splenic rupture was chosen, as there was no active contrast extravasation on the CECT scan. Hourly vitals were checked, and sixth hourly monitoring of hemoglobin, hematocrit, platelets, and blood gas was done. He was transfused blood products accordingly to maintain his hemoglobin and platelet counts. The massive transfusion protocol was not followed for this patient.

**Table 1:** Blood investigations "-": Blood investigation was not done on that day; INR: International normalized ratio; APTT: activated partial thromboplastin time; AST: aspartate transaminase; ALT: alanine transaminase; IU/L: International units/ liter; ng: nanogram; mg: milligram; dL: deciliter; g: gram

	Day 1	Day 2	Day 3	Day 4	Day 5	Day 6	Day of Discharge	Reference Range
Hemoglobin (g/dL)	13.7	10.5	9.2	8.1	7.7	8.4	10.3	13.2 - 16.6
Total count (10^3/microliter)	4.96	9.31	4.97	-	-	-	-	4 - 11
Platelet (10^3/microliter)	42	44	96	104	110	137	161	150 - 450
Packed cell volume	40.6%	-	-	-	-	-	31.6	40% - 54%
Prothrombin time (seconds)	12.4	-	-	11.5	-	-	-	11 - 13.5
INR	1.03	-	-	0.96	-	-	-	0.8 - 1.1
APTT (seconds)	37.9	-	-	32.9	-	-	-	25 - 35
Total bilirubin (mg/dL)	0.31	-	-	-	-	-	-	0.1 - 1.2
AST (U/L)	835	-	-	-	-	194	-	5 - 40
ALT (U/L)	432	-	-	-	-	241	-	7 - 56
Fibrinogen (mg/dL)	223	-	-	-	-	-	-	200 - 400
Serum ferritin (ng/mL)	11368.9	-	-	-	-	-	-	30 - 400
Serum triglycerides (mg/dL)	187	-	-	-	-	-	-	< 150

**Figure 2:**
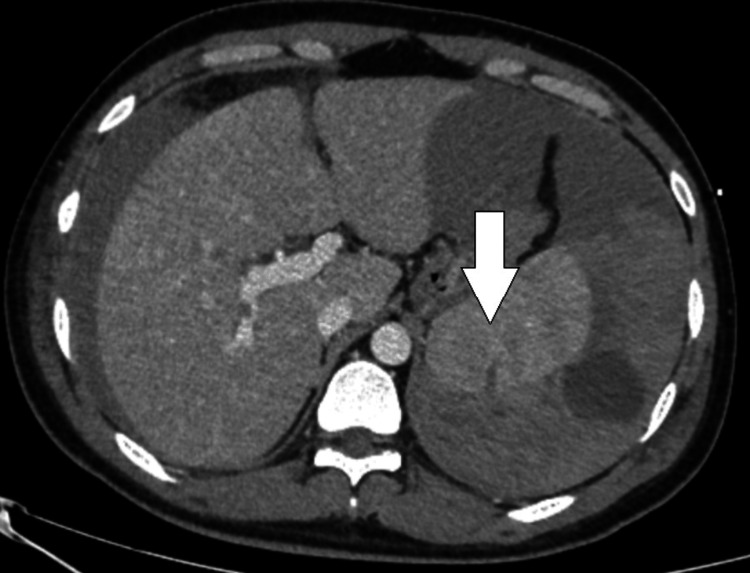
CECT abdomen on day 4, showing a decrease in the size of the hematoma CECT: Contrast-enhanced computed tomography

The platelet counts gradually improved from 42,000 per microlitre on day 1 to 104,000 per microlitre on day 4. A repeat abdominal scan was performed on day 4, showing a decrease in the size of the hematoma (Figure 2). By day six, the patient was weaned off ICU care and transferred to the general ward, remaining clinically and hemodynamically stable with recovery from dengue shock syndrome.

Case 2

A 23-year-old man with no significant past medical history presented with a four-day history of fever and headache, vomiting for two days, and breathlessness for one day. He tested weakly positive for dengue IgM and had thrombocytopenia (platelet count: 68 × 10³/μL) at an outside hospital. The patient was brought to our hospital unresponsive. He was directly triaged to the red zone of the Emergency department. Cardiopulmonary resuscitation (CPR) was initiated per ACLS protocol. In the first two rhythm checks, asystole was noted, and the subsequent 4 rhythm checks showed pulseless electrical activity. IV adrenaline was administered every 3-5 minutes. Return of spontaneous circulation (ROSC) was achieved after six cycles. Post-ROSC, the patient was intubated and resuscitated with IV fluids as he was dehydrated. He was started on vasopressors due to worsening shock.

A nasogastric (Ryle’s) tube aspirate showed coffee ground material, and the patient exhibited progressive metabolic acidosis and severe shock. Severe dengue hemorrhagic shock was suspected, and the management was started in line with dengue hemorrhagic shock. Due to hemodynamic instability, need for respiratory support, and intensive monitoring, the patient was admitted to the critical care unit for further management.

**Figure 3:**
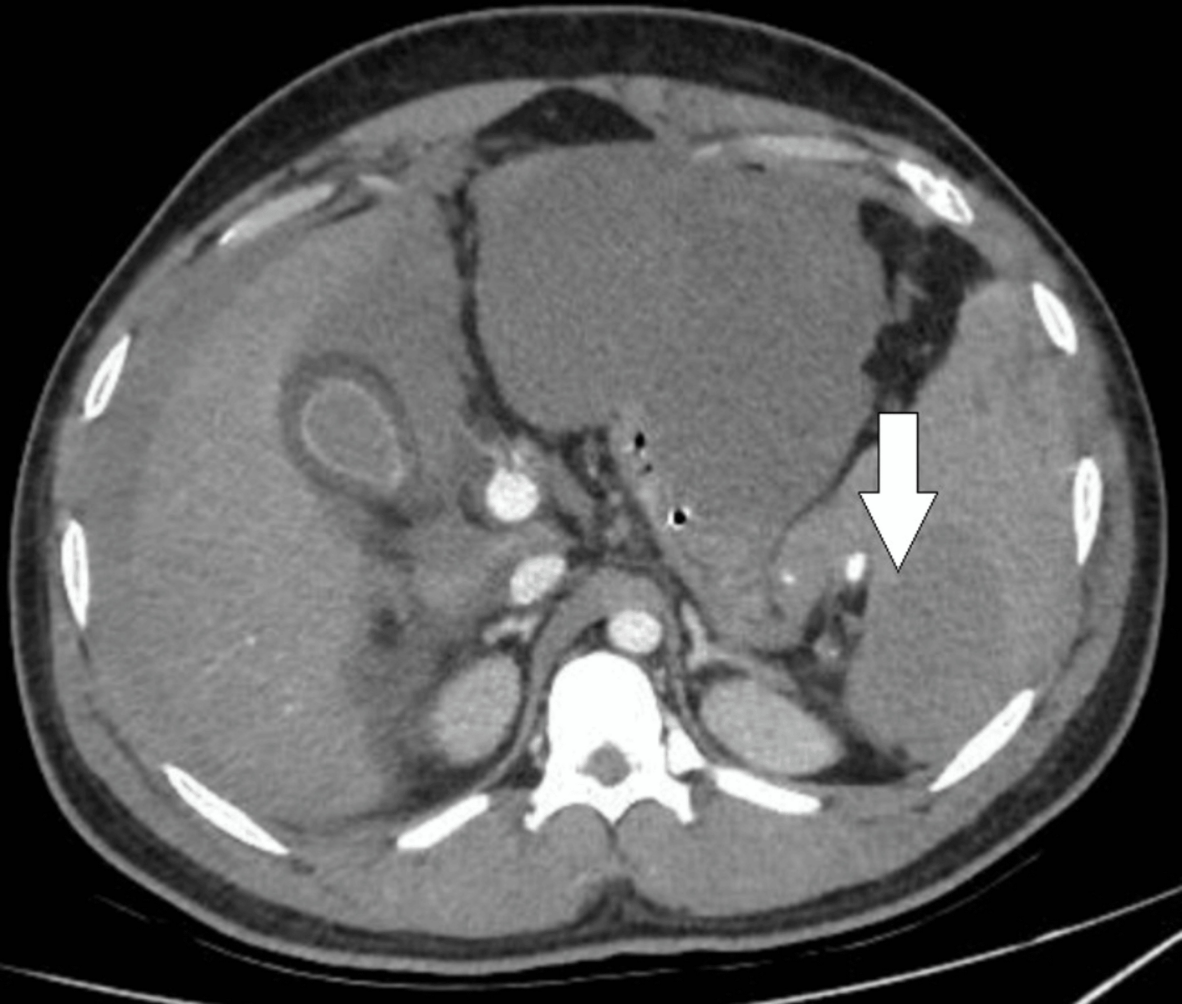
CECT abdomen on day 2 CECT: Contrast-enhanced computed tomography

**Figure 4:**
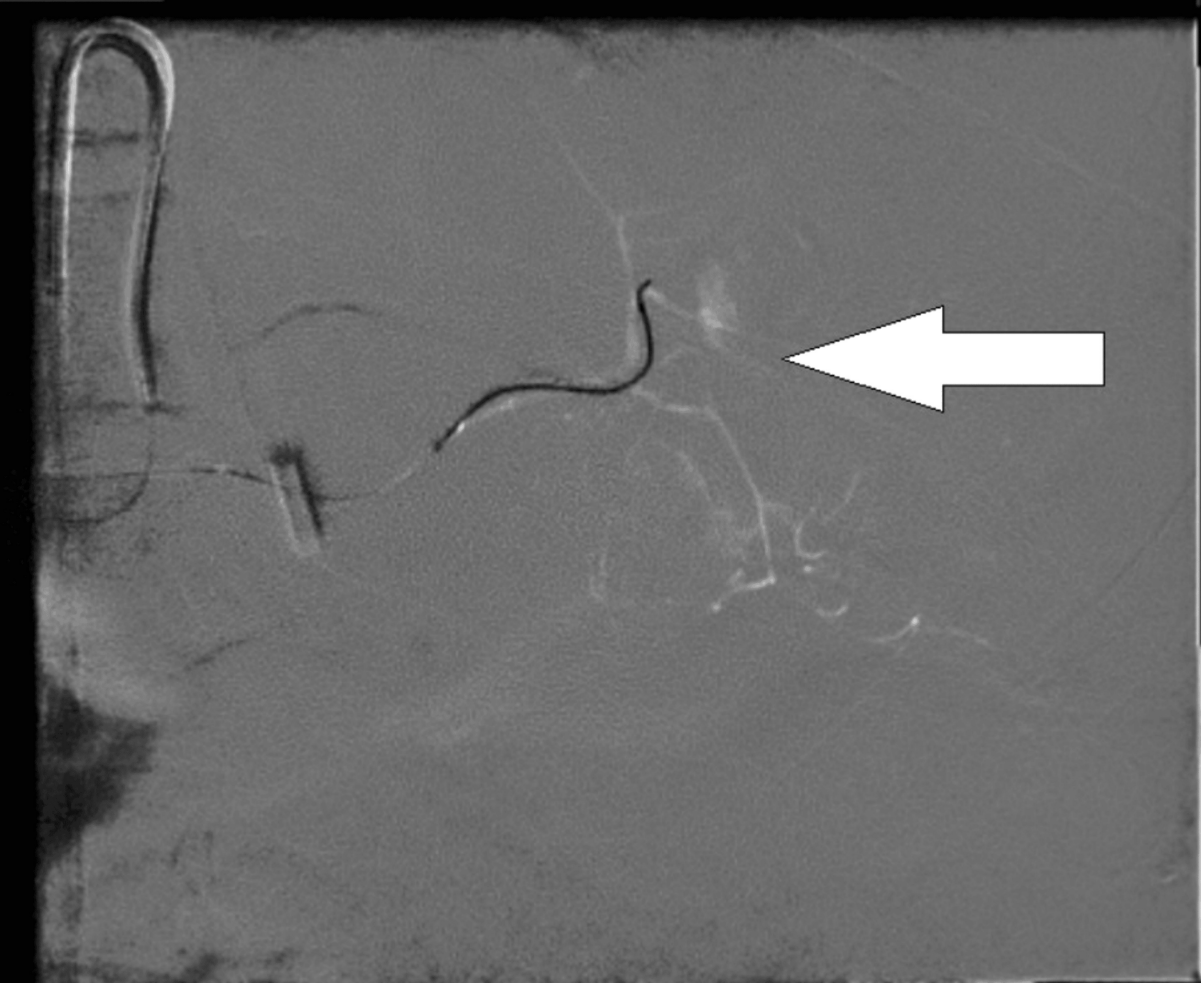
Angio-embolization of the splenic artery

In the ICU, lung-protective ventilation was continued along with fluid resuscitation, massive transfusion of blood products, vasopressor therapy, and continuous renal replacement therapy. POCUS revealed free fluid in the abdomen. Definitive radiological investigation was deferred on day one as the patient was too critical to be shifted for investigation. On day two of ICU stay, a CECT scan of the abdomen demonstrated active, progressive arterial contrast extravasation in the gastrosplenic region with moderate hemoperitoneum, likely arising from splenic arterial branches, along with a non-enhancing loculated fluid collection along the greater curvature of the stomach (Figure 3). Splenic artery angioembolization was performed to control ongoing hemorrhage (Figure 4).

The patient's laboratory investigations revealed a worsening AST/ALT level suggestive of shock liver/ dengue hepatitis (Table 2). Though the patient's lab values did not categorize into overt disseminated intravascular coagulation, the corresponding parameters were worsening. These findings were considered to be a part of the multi-organ dysfunction of the patient's illness.

**Table 2:** Blood investigations "-": Blood investigation was not done on that day; INR: International normalized ratio; APTT: activated partial thromboplastin time; AST: aspartate transaminase; ALT: alanine transaminase; IU/L: International units/ liter; ng: nanogram; mg: milligram; dL: deciliter; g: gram

	Day 1	Day 2	Day 3	Reference Range
Hemoglobin (g/dL)	7.8	4.3	6.8	13.2 - 16.6
Total count (10^3/microliter)	20.84	-	18.35	4 - 11
Platelet (10^3/microliter)	104	29	55	150 - 450
Packed cell volume	26.7%	14.4	-	40% - 54%
Prothrombin time (seconds)	17.9	-	25.6	11 - 13.5
INR	1.51	-	2.18	0.8 - 1.1
APTT (seconds)	109.6	-	60.8	25 - 35
Total bilirubin (mg/dL)	0.31	-	-	0.1 - 1.2
AST (U/L)	4002	-	14818	5 - 40
ALT (U/L)	3749	-	4523	7 - 56
Fibrinogen (mg/dL)	123	-	153	200 - 400
Serum ferritin (ng/mL)	40001	-	-	30 - 400
Serum triglycerides (mg/dL)	187	-	-	< 150
Serum Urea (mg/dL)	53	41	-	7 - 20
Serum creatinine (mg/dL)	2.76	2.79	-	0.6 - 1.3

Despite aggressive supportive measures, the patient’s shock worsened, and he ultimately succumbed to his illness on day 3 of hospitalization.

## Discussion

Dengue is a dynamic illness characterized by three phases: the acute febrile phase (1-3 days), the critical phase (4-6 days), and the recovery phase (beyond five days) [4]. Approximately 5-10% of patients may progress to the critical phase after the third to fourth day of illness, which is marked by vasculopathy, coagulopathy, and shock. Hemorrhagic manifestations can occur during both the febrile and afebrile phases, commonly involving the skin, mucosal surfaces, gastrointestinal tract, and genitourinary tract [3].

Atraumatic pathological rupture of the spleen, although rare, carries a significant risk of morbidity and mortality. The overall incidence of atraumatic splenic rupture ranges between 0.1% and 0.3%, with variation depending on the specific etiological factor. Common pathological causes include neoplastic diseases, infections, inflammatory conditions, drug-induced effects, and mechanical disorders, which together account for 93% of atraumatic splenic rupture cases, compared to 7% in an idiopathic normal spleen. The overall mortality rate is approximately 12.2%, with splenomegaly, age over 40 years, and neoplastic disorders identified as high-risk factors [9].

The World Society of Emergency Surgery (WSES) classifies splenic injury according to anatomical and hemodynamic status into mild (WSES Class I), moderate (Class II and III), and severe (Class IV) categories (Table 3) [10]. This classification aids in guiding management strategies.

**Table 3:** WSES classification of Splenic injury Source: [10]

WSES Class	Injury Description	Hemodynamic Status
Class I (Mild)	Minor splenic injuries without major vascular involvement	Hemodynamically stable
Class II (Moderate)	Moderate splenic injuries with limited vascular involvement	Hemodynamically stable
Class III (Moderate)	More extensive splenic injuries, possibly with segmental vascular injury	Hemodynamically stable with potential signs of instability
Class IV (Severe)	Severe splenic injuries with major vascular involvement or a shattered spleen	Hemodynamically unstable

Management of splenic subcapsular hematoma remains controversial. While early splenectomy has traditionally been advocated to prevent rupture and complications, recent evidence suggests that many cases may regress with conservative management or splenic artery embolization [9,11,12].

In hemodynamically stable patients, non-operative, organ-preserving approaches can be successful, provided there is careful patient selection and access to appropriate monitoring and interventional radiology or surgical support.

In our first case, the patient was hemodynamically stable corresponding to Class II of WSES classification of splenic injury. He was managed successfully with close monitoring and multidisciplinary team involvement. Conversely, angioembolization serves as a salvage therapy in moderate to severe cases where hemodynamic instability and severe coagulopathy preclude surgical intervention. Our second patient presented with severe shock, coagulopathy, and multi-organ dysfunction and underwent splenic artery embolization as a source control measure, as he was considered too unstable for surgery corresponding to Class IV in the WSES classification of splenic injury [9,10].

In both these cases, the splenic rupture was diagnosed during the critical phase of the illness (days 4-6) [4].

The primary objective in managing bleeding in coagulopathic patients is prompt control of the bleeding source to achieve hemodynamic stability. In atraumatic splenic rupture, it is critical to address the underlying pathology precipitating the event. In both our cases, dengue-associated coagulopathy was the key etiologic factor. Although both patients presented during the critical phase of dengue illness, their clinical courses diverged-one toward recovery and the other toward deterioration. Our decisions regarding conservative versus interventional management were therefore guided not only by baseline patient characteristics but also by their dynamic clinical progression in the ICU setting.

This case report is based on a single-center experience of managing two cases, and hence, generalizability is limited.

## Conclusions

Management of splenic subcapsular hematoma in thrombocytopenic and coagulopathic patients warrants a high index of suspicion and early evaluation. A careful, individualized approach is essential. The decision regarding the urgency and type of intervention should be guided primarily by the patient’s clinical condition, hemodynamic stability, and coagulation status rather than imaging findings alone. Premature or aggressive intervention in a bleeding, thrombocytopenic patient may lead to adverse outcomes. This type of situation needs a multidisciplinary approach with the readiness to switch to an interventional or surgical approach from a conservative approach or vice versa, depending on disease progression. Therefore, a balanced strategy that thoughtfully integrates both conservative and interventional options is essential to optimize patient outcomes.

## References

[1] Ross TM (2010). Dengue virus. Clin Lab Med.

[2] (2025). Dengue. https://www.who.int/news-room/fact-sheets/detail/dengue-and-severe-dengue.

[3] (2023). National Guidelines for Clinical Management of Dengue Fever. https://ncvbdc.mohfw.gov.in/Doc/National%20Guidelines%20for%20Clinical%20Management%20of%20Dengue%20Fever%202023.pdf.

[4] Bhalla A, Singh H, Suri V (2024). ISCCM position statement: management of severe dengue in intensive care unit. Indian J Crit Care Med.

[5] Dronamraju SS, Gaidhane SA, Mahalaqqa KN, Gaidhane AM, Andhale AG, Quazi ZS (2021). Splenic artery embolization in subcapsular splenic hematoma secondary to dengue hemorrhagic fever. J Glob Infect Dis.

[6] Radwan I, Magdy Khattab M, Mahmoud AR (2019). Systematic review of spontaneous splenic rupture in dengue-infected patients. Rev Med Virol.

[7] Padyana M, Gopaldas JA, Karanth S (2020). A stitch in time dengue with spontaneous splenic rupture. Radio Infect Dis.

[8] (2025). National Early Warning Score (NEWS) 2. https://www.rcp.ac.uk/improving-care/resources/national-early-warning-score-news-2/.

[9] Renzulli P, Hostettler A, Schoepfer AM, Gloor B, Candinas D (2009). Systematic review of atraumatic splenic rupture. Br J Surg.

[10] Coccolini F, Montori G, Catena F (2017). Splenic trauma: WSES classification and guidelines for adult and pediatric patients. World J Emerg Surg.

[11] Bhaskar E, Moorthy S (2012). Spontaneous splenic rupture in dengue fever with non-fatal outcome in an adult. J Infect Dev Ctries.

[12] Xia FF, Li QK, Zhang Y (2024). Comparison of splenic embolization and splenectomy for traumatic splenic rupture: a meta-analysis. Minim Invasive Ther Allied Technol.

